# Supplementary material for: Phosphoglucomutase A-mediated metabolic adaptation is essential for antibiotic and disease persistence in Mycobacterium tuberculosis
Source: mSystems. 2025 Jun 30;10(7):e00420-25. doi: 10.1128/msystems.00420-25 (PMC12282177; doi:10.1128/msystems.00420-25)
Supplement: Supplemental material — Figures S1 to S6 and Tables S1 to Table S4. [file msystems.00420-25-s0001.pdf]

Supplementary Figures and Tables

Title: Phosphoglucomutase A mediated metabolic adaptation is essential for antibiotic and disease persistence in *Mycobacterium tuberculosis*

Taruna Sharma[1,2] *et. al*.

[1]Mycobacterial Pathogenesis Laboratory, Centre for Tuberculosis Research, Translational Health Science and Technology Institute, Faridabad, Haryana, India.

[2]Jawaharlal Nehru University, New Delhi, India.

[3]Experimental Animal Facility, Translational Health Science and Technology Institute, Haryana, India.

[#]Correspondence-

Amit Kumar Pandey[1,3#] : amitpandey@thsti.res.in

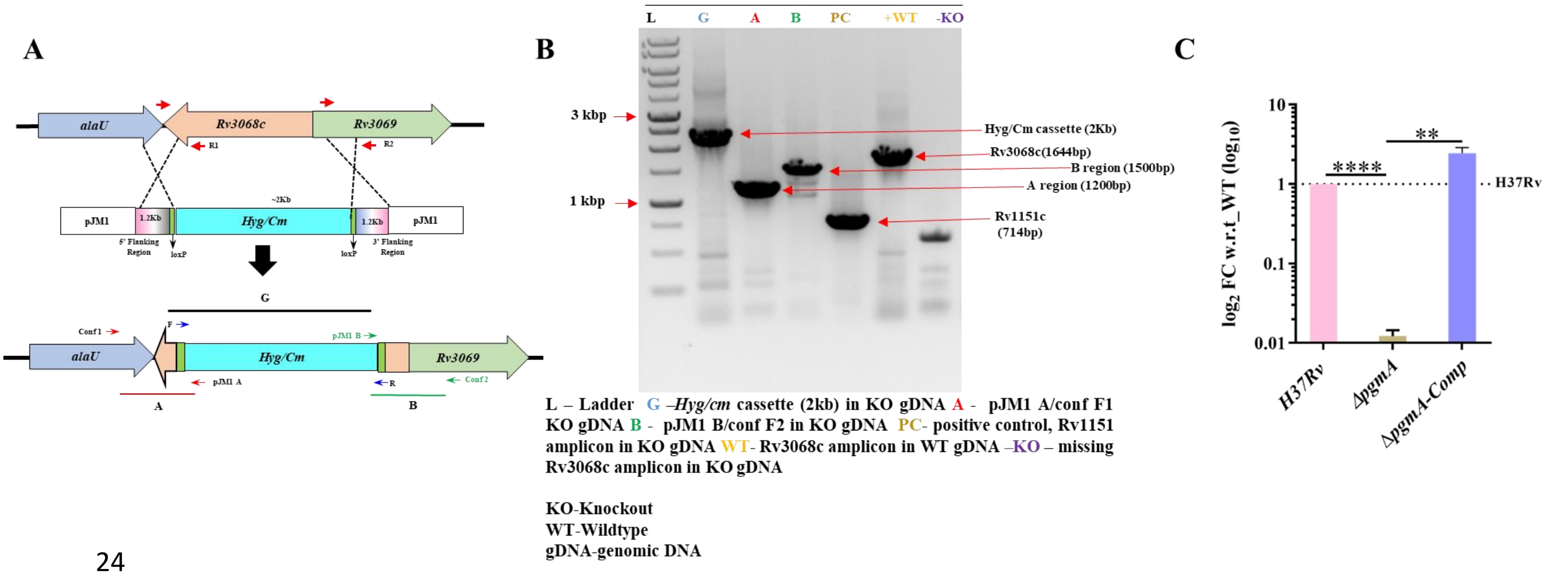

Supplementary Figure S1- Generation of Mtb *Rv3068c* deletion mutant (A) *Rv3068c* (*pgmA*) mutant generation strategy by RecET mediated genetic recombineering *(1)* (B) *Rv3068c* mutant confirmation by PCR (C) Expression level of *pgmA* gene in *ΔpgmA* and *ΔpgmA::pgmA* by RT-qPCR and quantified by $\Delta\Delta C_T$ method. Statistical significance of (C) was determined using unpaired, non-parametric two-tailed t-test $^{**}P \leq 0.005$ and $^{****}P \leq 0.00005$. Data represent mean ± SEM (standard error mean) for technical triplicates.

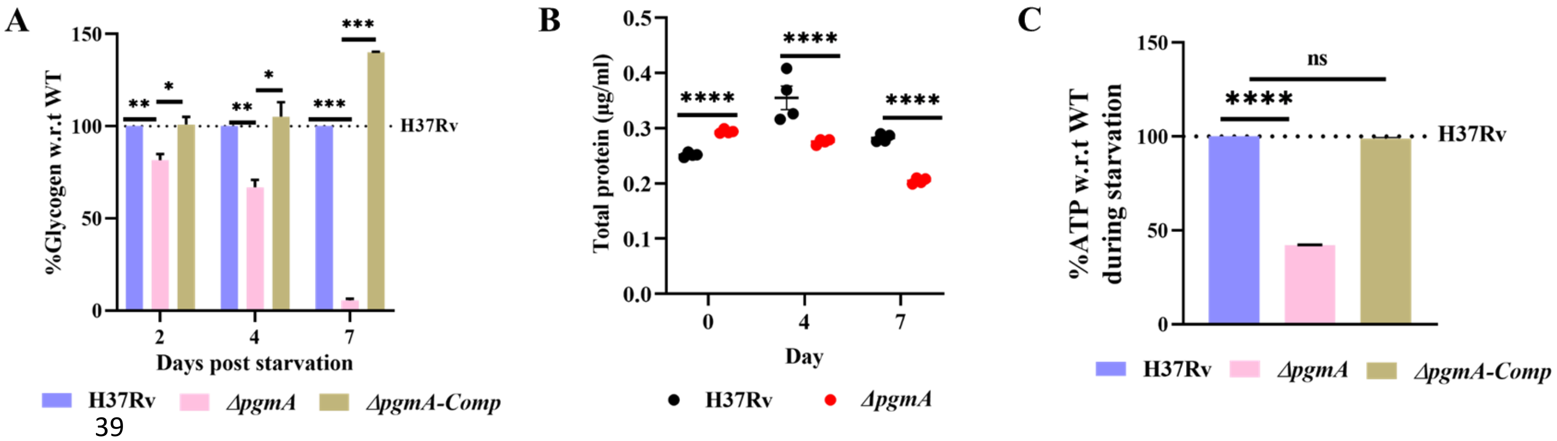

Supplementary Figure S2- Deletion of *pgmA* leads to impaired glycogen levels and overall biomass. (A) Glycogen estimation using colorimetric based EnzyChrom glycogen assay kit (Bioassay Systems) under glucose free condition at Day 2, 4 and 7. (B) BCA estimation in PBST. (C) ATP measurement of H37Rv and *ΔpgmA* under starvation condition using BacTiter-Glo™ microbial cell viability assay kit (Promega). Statistical significance of (A) to (C) was determined using unpaired, non-parametric two-tailed t-test *P ≤ 0.005, **P ≤ 0.005 and ****P ≤ 0.00005. Data represent mean ± SEM (standard error mean) for technical triplicates.

S.No	Lipid	Solvent system
1	Mycolic acid	Hexane: ethyl acetate (95:5 v/v)
2	PDIM	Petrolium ether: diethyl ether (9:1v/v)

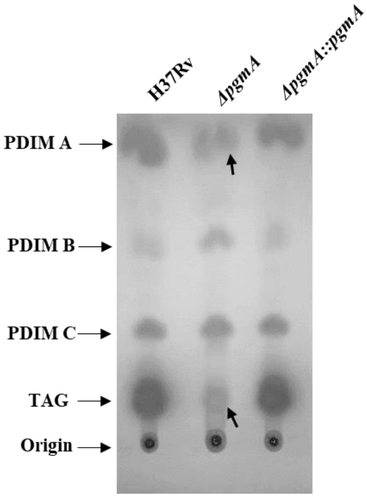

C

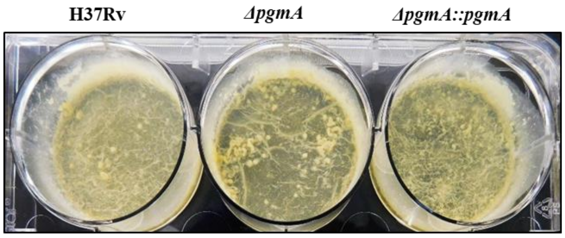

D

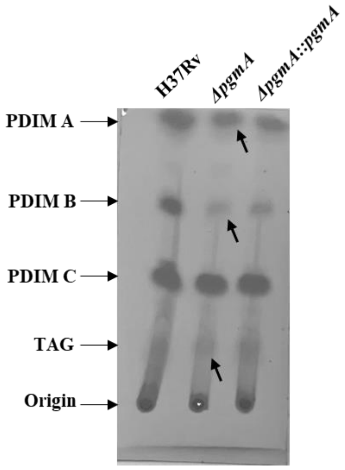

E

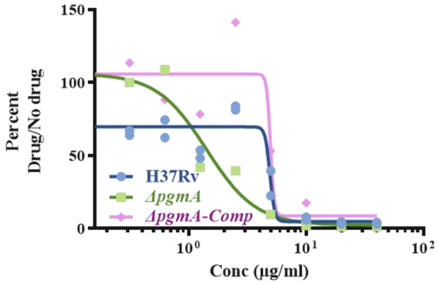

	H37Rv	ΔpgmA	ΔpgmA-Comp
IC50	~ 4.913	1.415	~ 4.971

Supplementary Figure S3- Deletion of *pgmA* impairs cellular integrity. (A) Solvent system used for the separation of individual lipids by TLC from total lipids. (B) 1D separation of apolar fraction of total lipids by TLC. (C) *ΔpgmA* showing defect in biofilm formation. (D) TLC separation of apolar fraction of total biofilm lipids showing defects in PDIMs and TAGs. (E) Dose response curve of vancomycin in H37Rv, *ΔpgmA* and *ΔpgmA::pgmA* with IC50 values depicted in the table under the figure. Abbreviations: PDIMs= phthioceroldimycocerosates, TAGs=triacylglycerols, TLC= thin layer chromatography.

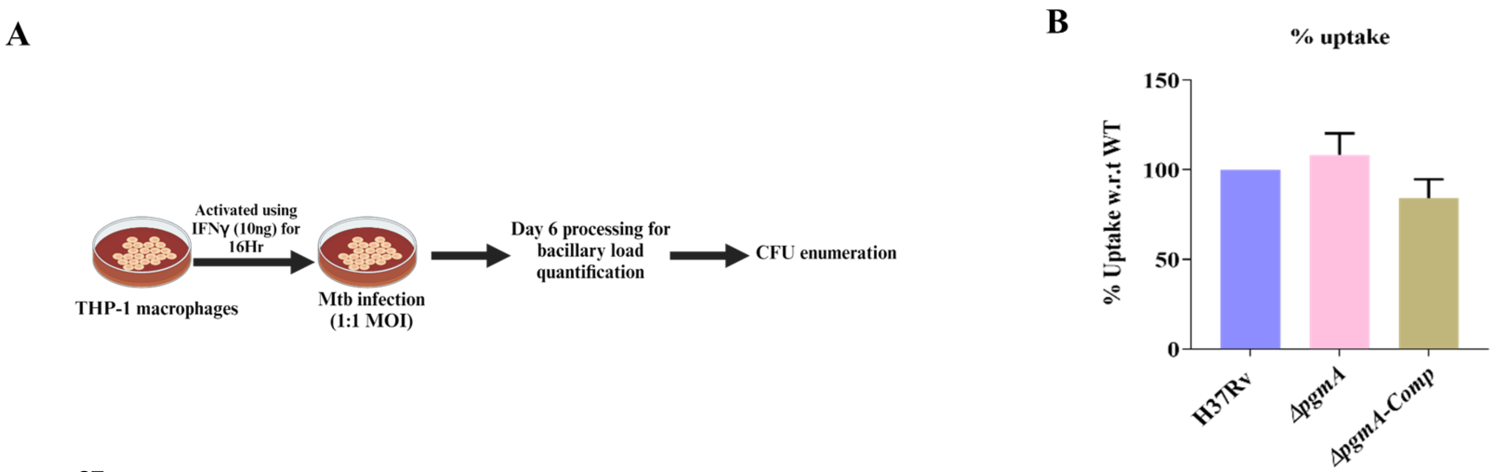

Supplementary Figure S4- THP-1 macrophages infected with Mtb (A) Diagrammatic representation of an *ex-vivo* growth kinetics assay employing activated THP-1 macrophages. The macrophages were activated by treatment with IFNγ (10 ng/ml) for 16 hours after which they were infected with MOI 1:1). (B) %uptake of H37Rv, *ΔpgmA* and *ΔpgmA-Comp* by THP-1 macrophages post 4 Hr infection.

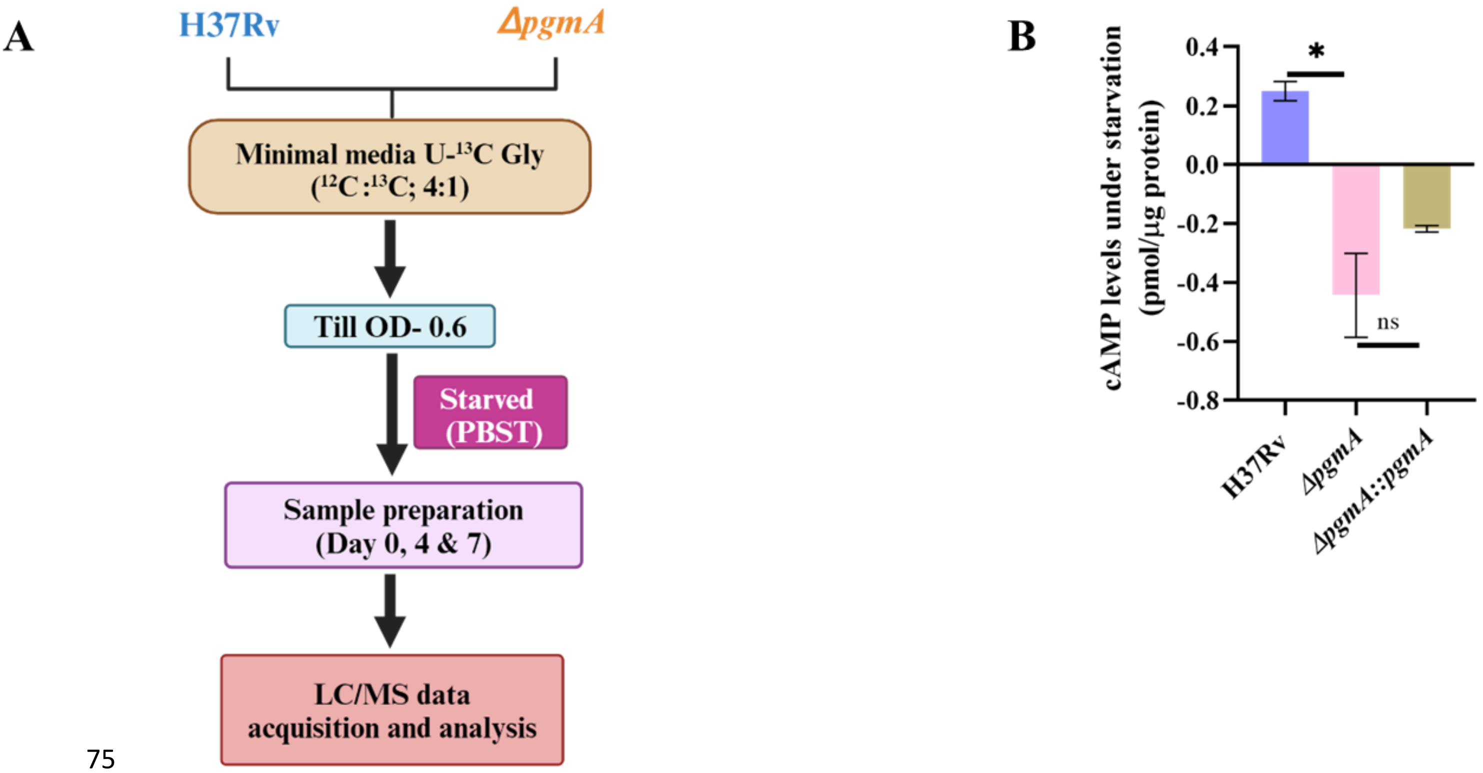

Supplementary Figure S5- *pgmA*-mediated regulation of carbon flux is crucial for the survival of Mtb under nutrient stress. (A) Diagram illustrating the process of isotopic labeling with ^{13}C, wherein Mtb strains were initially cultured in minimal media containing U-^{13}C glycerol (0.1%, ^{13}C /^{12}C, ¼:v/v). Samples were collected on days 4 and 7 post-starvation and processed for liquid chromatography and mass spectrometry (LC/MS) data acquisition. (B) Under-representation of cAMP levels in *ΔpgmA* under starving conditions measured using direct immunoassay based kit. Statistical significance was determined using unpaired, non-parametric two-tailed t-test $*P \leq 0.005$, ns=non-significant. Data represent mean ± SEM (standard error mean) for technical triplicates.

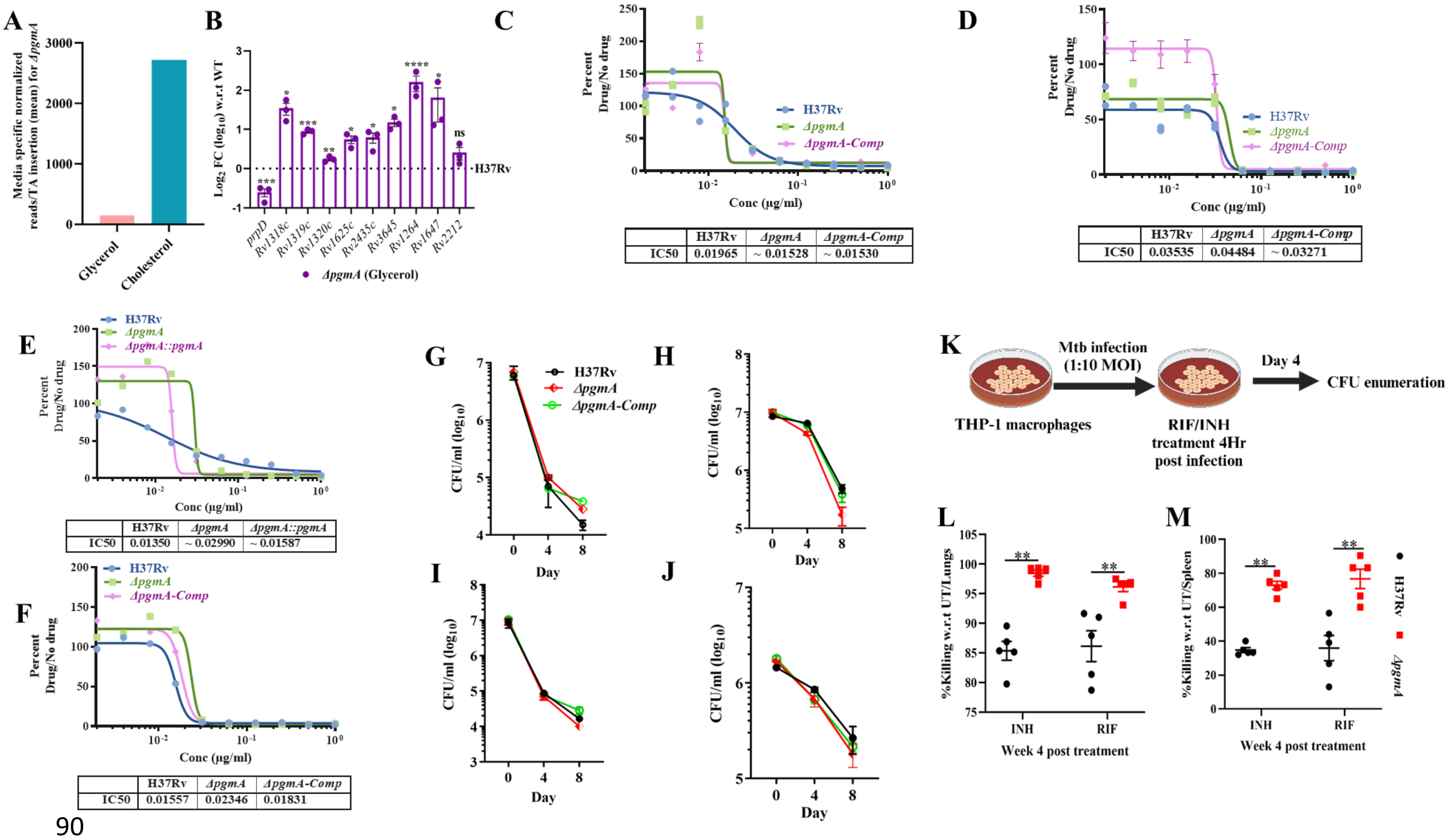

Supplementary Figure S6- Data showing carbon source specific drug susceptibility of *ΔpgmA*. (A)TraSH data depicting transposon insertion mutant of *pgmA* showing ~18-fold over-representation in cholesterol-rich media compared to glycerol-rich media. This calculation was based on the mean number of reads detected per TA insertion site as shown by Sassetti and colleagues (*2*). (B) The expression levels of adenylate cyclases under glycerol-specific media conditions in *ΔpgmA* with respect to (w.r.t) H37Rv. Bioluminescence-based assays utilized to determine the MICs of RIF and INH in 7H9 media (C and D) and glycerol media (E and F) respectively. IC50 values are mentioned below the dose response curve under respective media conditions. In (G) to (J), time kill assay was conducted *in-vitro* using RIF and INH, with (G) and (I) representing 7H9 media, and (H) and (J) representing glycerol media, respectively. (K) Schematic depiction of the *ex-vivo* drug susceptibility assay conducted in the

THP1 cell line is as follows: THP1 cells were infected at 1:10 MOI with subsequent drug treatment administered after 4 hours of infection. In (L) and (M), Percentage killing with respect to (w.r.t) untreated group was evaluated in lung (L) and spleen (M) of infected mice at week-4 post-treatment. Statistical significance in (B), (G) to (J) and (L) to (M) was determined using unpaired, non-parametric, two-tailed t-test $^*P \leq 0.005$, $^{**}P \leq 0.005$, $^{***}P \leq 0.0005$ and Mann-Whitney test $^{**}P \leq 0.008$ respectively. Data represent mean ± SEM (standard error mean) for technical triplicates. Abbreviation: MOI= multiplicity of infection, MIC= minimum inhibitory concentration

Reference:

1. K. C. Murphy, K. Papavinasasundaram, C. M. Sassetti, in *Mycobacteria Protocols*, T. Parish, D. M. Roberts, Eds. (Springer New York, New York, NY, 2015), pp. 177-199.
2. J. E. Griffin *et al.*, High-resolution phenotypic profiling defines genes essential for mycobacterial growth and cholesterol catabolism. **7**, e1002251 (2011).

Supplementary tables

S. No.	Strain	Marker	Feature	Reference
1.	*E. coli* XL1Blue			For cloning
2.	*M. tuberculosis* H37Rv		Wild type *M. tuberculosis*.	Kind gift from Christopher M. Sassetti
3.	*ΔpgmA*	Hyg^r,	*Rv3068c*, *pgmA* deletion mutant	This work
4.	*ΔpgmA-Comp*	Kan^r	*ΔpgmA* mutant expressing pMV261:*pgmA* and *pJEB408:pgmA* plasmid	This work
5.	Rv:: *Lux*	Zeo^r,	Tweety int site, Luciferase reporter	This work
6.	*ΔpgmA::Lux*	Hyg^r,Zeo^r ,	Tweety int site, Luciferase reporter	This work
7.	*ΔpgmA Comp:: Lux*	Kan^r, Zeo^r,	Tweety int site, Luciferase reporter	This work
8.	Rv: pMV261TagGFP	Hyg^r,	GFP expressing Rv-WT	This work
9.	*ΔpgmA*: pMV261TagGFP	Hyg^r,	GFP expressing in *ΔpgmA* strain	This work
10.	*ΔpgmA Comp*: pMV261TagGFP	Kan^r, Hyg^r	GFP expressing in *ΔpgmA Comp* strain	This work
	**Cell line**	**Description**		**Source**
1.	THP-1	Human monocyte cell line		NCCS,Pune, India

Supplementary Table S1: Bacterial strains and cell lines used in the study

Primers	Sequence	Remarks
pgmA-F1	GCCTTAATTAACCAGAAACCTCCCCGCC	*pgmA* cloning and mutant confirmation
pgmA -R1	GACACTAGTGACGAGGTCTTCGGGCTG	
pgmA -F2	ATTCTCGAGTCCTTCCGTGGACCGCAAC	
pgmA -R2	ATTGGGCCCCGACCTACTACTTCAATGACCTCG	
pgmA -conf1	CTCGGACAGGCTCAGCGATT	
pgmA -conf2	GTCCACGCGACCCAGC	
Rv1264-F	GGGCTGAATCCCGACCAAGT	*M. tuberculosis* Adenylate cyclase qPCR primers
Rv 1264-R	CCAGCAGCGGCACGATCT	
Rv 1647-F	CGGCAAACCCGGAGGTGA	
Rv 1647-R	TGATCCCGTCGCCCAGC	
Rv 2212-F	GAGGACGGCGCATTCGG	
Rv 2212-R	CGCCTTCAAAGTAGGTTCGTGC	
Rv1318-F	GTTCTGGGGTGTTGGCACG	
Rv1318-R	CGGCGAACGGGCGCAAT	
Rv1319-F	AGCGTCCATACGGTGGGC	
Rv1319-R	GCGAACTCGGTGAACAAGTAGC	
Rv1320-F	GTTTGCGCTTCGCCCGATG	
Rv1320-R	TGGTGCCCACAGGATCAGC	
Rv1625-F	CTGGCTCGCTGTCGTGGTAA	
Rv1625-R	CGTCGGTGCCCACATCC	
Rv2435c-F	ACTTTACCTGGATTACCGACTTCAAGC	
Rv2435c-R	AATCGGACCGCATCAGACTGTC	
Rv3645-F	CACTCCGTGGCCGCTGT	
Rv3645-R	GGTGAGCAGGTTGTCGCG	

Supplementary Table S2: Primers used in the study

Functional category	Up regulated genes	Down regulated genes	Total Up regulated genes	Total down regulated genes
**Starvation/late stationary phase/NRP**	*Rv 3290, Rv2891, Rv0251c, Rv3288c, Rv2699c, Rv0120c, Rv2632c, Rv2161c, Rv1288, Rv1834, Rv0458*	*Rv2665*	11	1
**Immune modulation**	*Rv1804c, Rv2353c, Rv1813c*	*Rv2307c, Rv1146 (mmpl13b)*	3	2
**Transcription factors**	-	*Rv3160c, Rv0792c, Rv2327, Rv2250c, Rv1499, Rv3124(moaR1), Rv0232, Rv0144, Rv0081, Rv0818*	0	10
**Transposon/insertion elements**	-	*Rv3186, Rv1313c, Rv0795, Rv1036c, Rv1585c, Rv0920c, Rv2651c, Rv3844, Rv2812, Rv1573, Rv1586c, Rv1580c*	0	12
**Membrane protein/permease/lectins/Cell wall/peptidoglycan**	*Rv2340c, Rv1004c, Rv1999c*	*Rv0666, Rv0488, Rv0219, Rv1382, Rv2254c, Rv0680c, Rv2307c, Rv0463, Rv0048c, Rv2293c, Rv0289 (espG3), Rv2723, Rv2686c (ABC transporter), Rv2325c, Rv2403c (lppR), Rv1986, Rv2643 (arsC), Rv3454, Rv0473, Rv3821, Rv1541c, Rv1217c (ABC transporter), Rv1146 (MmpL13b), Rv1038c (esxJ), Rv1793 (esxN), Rv1914c, Rv2620c, Rv0588 (yrbE2B), Rv0008c, Rv0584*	3	30
**Antibiotic resistance**	*Rv1930c, Rv3614c*	*Rv0095c, Rv0856, Rv0071*	2	3
**Intermediary metabolism/respiration/DNA synthesis & repair**	*Rv0356c, Rv0223c, Rv1393c, Rv1834 (lipZ)*	*Rv1571, Rv0791c, Rv0693(mftC), Rv0331, Rv2622, Rv0591 (mce2C), Rv3352c, Rv0166 (fadD5), Rv2381c (mbtD), Rv0812, Rv0694(mftD), Rv1939, Rv3703c, Rv1204c, Rv3701c, Rv2678c(hemE), Rv1089A (celA2a), Rv0322 (udgA), Rv1538c (ansA), Rv1203c, Rv3685c (cyp135), Rv3026c, Rv2435c (adenylyl cyclase), Rv0594 (mce2F), Rv2153c (MurG), Rv1888c, Rv3588c (canB), Rv0816c (thiX), Rv2423, Rv0762c, Rv3468c, Rv1853, Rv0601c, Rv1263 (amiB2), Rv0470c (pcaA), Rv1681 (moeX), Rv2934 (ppsD), Rv1570 (bioD), Rv1302 (rfe), Rv3556c (fadA6), Rv3436c (glmS), Rv0614 Rv0766c (cyP123), Rv3010c (pfkA), Rv0674, Rv0224c, Rv3322c, Rv1820 (ilvG), Rv3097c (lipY), Rv1319c (adenylyl cyclase), Rv2384 (mbtA), Rv0202c (mmpl11), Rv2949c, Rv0695 (mftE), Rv2840c, Rv0587 (yrbE2A), Rv2948c (fadD2), Rv2932 (ppsB), Rv0521, Rv0673 (echA4), Rv0726c, Rv2065 (cobH), Rv0469, Rv1847, Rv0539, Rv0189c, Rv3176c (mesT), Rv3312c, Rv0197, Rv2982c (gpdA2), Rv3473c (bpoA, Rv3581c (ispF), Rv3470c, Rv0370c, Rv2766c (FabG5), Rv3704c (gshA), Rv2505c (fadD35), Rv0096, Rv1546, Rv0968, Rv2737c, Rv3466, Rv1850 (ureC), Rv1641 (infC), Rv3202c, Rv0629c (recD), Rv1537 (dinX), Rv3201c, Rv2132, Rv2836c, Rv2885c, Rv2404c (lepA), Rv0269c, Rv2839c (infB), Rv3394c, Rv1003, Rv2755c, Rv3687c (rsfB), Rv2948c (fadD22)*	4	97
**TA system**	*Rv3357*	*Rv2758c, Rv0626, Rv0596c, Rv0623 (vapB30), Rv2862c (vapB23), Rv2827c, Rv2274c (mazF8), Rv1990*	1	8
**PE-PGRS**	*Rv3590*	*Rv0832, Rv1452c, Rv2107*	1	3
**Conserved hypothetical**	*Rv1670*	*Rv0863, Rv2489c, Rv3098c, Rv3647c, Rv3142c, Rv1684, Rv3163c, Rv2023c, Rv3439c, Rv0378, Rv2134c, Rv2722, Rv0610c, Rv2086, Rv1191, Rv2183c, Rv1682, Rv2566, Rv3771c, Rv2433c, Rv2076c, Rv2807, Rv2644c, Rv1060, Rv0036c, Rv1518, Rv0021c, Rv1116A*	1	28

Supplementary Table S3: Functional separation of up and down regulated DEGs obtained in RNAseq analysis exclusively in *ΔpgmA* using Go-ontology

Subsystems	Up-regulated_genes	Down-regulated_genes	Total_Up	Total_Down	Total
**Membrane Metabolism**	-	*Rv0166, Rv2381c, Rv2384, Rv2505c (fadD35), Rv2932, Rv2934, Rv2948c (fadD2), Rv2949c, Rv2293c, Rv1546,*	0	10	10
**Cofactor and Prosthetic Group Biosynthesis**	-	*Rv0202c, Rv1570, Rv2065, Rv2678c, Rv3322c*	0	5	5
**Fatty Acid Metabolism**	-	*Rv0166, Rv0673, Rv2505c, Rv2948c, Rv3556c*	0	5	5
**Translation/DNA synthesis & repair**	-	*Rv1850 (UreC), Rv1853 (ureD), Rv3202c (RecF), Rv2737c, Rv3466, Rv1641, Rv3202c, Rv0629c, Rv1537, Rv3201c, Rv2132, Rv2836c, Rv2885c, Rv2404c, Rv0269c, Rv2839c, Rv3394c, Rv1003, Rv2755c, Rv3687c*	0	20	20
**Valine, Leucine, and Isoleucine Metabolism**	-	*Rv0189c, Rv1820, Rv3470c, Rv3556c*	0	4	4
**Alanine, Aspartate, and Glutamate Metabolism**	-	*Rv1538c, Rv3704c*	0	2	2
**Glycolysis/Gluconeogenesis**	*Rv0458*	*Rv3010c (pfk)*	1	1	2
**Arabinogalactan biosynthesis**		*Rv1302, Rv3468c, Rv3436c (glmS)*	0	2	2
**Beta oxidation of unsaturated fatty acids**		*Rv0673, Rv3556c*	0	2	2
**Glycerophospholipid metabolism**		*Rv2982c, Rv3176c*	0	2	2
**Mycobactin biosynthesis**		*Rv2381c, Rv2384*	0	2	2
**Other Amino Acid Metabolism**	*Rv0223c*	*Rv1263*	1	1	2
**Pyruvate Metabolism**		*Rv0694, Rv3556c*	0	2	2
**Cholesterol degradation**		*Rv3556c*	0	1	1
**Degradation/Utilization/Assimilation**		*Rv3176c*	0	1	1
**Ergothioneine biosynthesis**		*Rv3703c*	0	1	1
**Folate Metabolism**		*Rv0812*	0	1	1
**Glycerolipid metabolism**	*Rv0458*	-	1	0	1
**L-alpha-aminoadipic acid (L-AAA) biosynthesis**	*Rv3290c*	-	1	0	1
**Lipid metabolism**		*Rv3097c (lipY), Rv2932 (ppsB), Rv2766c (FabG5)*	0	3	3
**Miscellaneous**		*Rv3588c*	0	1	1
**Mycolic acid pathway**	*Rv1288*	*Rv0470c (pcaA)*	1	1	2
**Nucleotide Salvage Pathway**	-	*Rv0322*	0	1	1
**Peptidoglycan Metabolism**	-	*Rv2153c*	0	1	1
**Polyprenyl Metabolism**	-	*Rv3581c*	0	1	1
**Redox Metabolism**	-	*Rv0021c, Rv0694 (mftC), Rv0693 (mftD), Rv0695 (mftE)*	0	4	4
**Respiration**	-	*Rv0766 (Cyp123 P450),*	0	1	1
**Metabolic signaling**	-	*Rv2435c (AC), Rv1319c (AC)*	0	2	2

Supplementary Table S4: Pathway analysis of up and down regulated metabolic genes obtained in RNAseq analysis.
